# Population-Specific Anatomical Variations in Premolar Root Canal Systems: A Cross-Sectional Cone-Beam Computed Tomography Study of Jamaican and Portuguese Subpopulations

**DOI:** 10.3390/dj13020050

**Published:** 2025-01-23

**Authors:** Jorge N. R. Martins, Sriteja Tummala, Sasidhar Nallapati, Duarte Marques, Emmanuel João Nogueira Leal Silva, João Caramês, Marco A. Versiani

**Affiliations:** 1 Faculdade de Medicina Dentária, Universidade de Lisboa, 1600-277 Lisboa, Portugal; duarte.marques@edu.ulisboa.pt (D.M.); carames@campus.ul.pt (J.C.); 2 Grupo de Investigação em Bioquimica e Biologia Oral, Unidade de Investigação em Ciências Orais e Biomédicas (UICOB), 1600-277 Lisboa, Portugal; 3 Centro de Estudo de Medicina Dentária Baseada na Evidência, 1600-277 Lisboa, Portugal; 4 LIBPhys-FCT UID/FIS/04559/2013, Faculdade de Medicina Dentária, Universidade de Lisboa, 1600-277 Lisboa, Portugal; 5 Instituto de Implantologia, 1070-064 Lisboa, Portugal; 6 Faculty of Medical Sciences, School of Dentistry, The University of the West Indies, Kingston JMAAW15, Jamaica; 7 Private Practice, Kingston JMAAW15, Jamaica; 8 Department of Endodontics, School of Dentistry, Grande Rio University, Rio de Janeiro 21210-623, Brazil; 9 Department of Endodontics, Rio de Janeiro State University, Rio de Janeiro 20550-013, Brazil; 10 Dental Specialty Center, Brazilian Military Police, Belo Horizonte 30350-190, Brazil

**Keywords:** anatomy, cone-beam computed tomography, endodontics, premolars, prevalence study, root canal treatment

## Abstract

**Background/Objectives:** The morphology of roots and root canals has a significant influence on the outcome of endodontic treatment. This study aimed to compare premolar root and root canal configurations between Central American and European subpopulations using cone-beam computed tomography (CBCT). **Methods:** This cross-sectional retrospective study analyzed CBCT scans of 2919 premolars from 561 patients: 197 from Jamaica (Central America) and 364 from Portugal (Europe). The analysis focused on the number of roots, root canal configurations, and the presence of C-shaped canals in mandibular premolars. Demographic factors such as age and sex were also considered. Statistical significance was determined using chi-square tests with a significance level set at *p* < 0.05. **Results:** Overall, premolars in Jamaican patients had more roots and root canals compared to those in Portuguese patients. Three-rooted configurations were more common in Jamaica, particularly in maxillary first premolars (5.5% vs. 2.9%, *p* < 0.05). Vertucci Type V configuration was more prevalent in the Central American subgroup, while Portuguese premolars were more likely to exhibit Types II and IV configurations. C-shaped canals were rare in both populations (*p* > 0.05). Males and younger patients showed higher frequencies of multi-root and multi-canal configurations (*p* < 0.05). **Conclusions:** This study reveals significant geographic and demographic differences in premolar root and root canal morphology between two Central American and European subpopulations. Premolars from Jamaica tend to exhibit more complex anatomical features compared to those from Portugal. These findings highlight the need for population-specific diagnostic and treatment strategies and demonstrate the value of CBCT in the preoperative diagnosis of complex anatomical variations.

## 1. Introduction

The morphology of roots and root canals has a significant influence on the outcome of endodontic treatment [1]. A thorough understanding of anatomical variations is essential for managing complex cases, especially in teeth with intricate root canal systems. Premolars are particularly recognized for their anatomical diversity, exhibiting variations in the number of roots, root canal configurations, and less common features such as C-shaped canals [2,3,4,5]. These complexities present clinical challenges, underscoring the importance of detailed anatomical knowledge for precise diagnosis and effective treatment planning.

Research has shown that root canal morphology varies significantly across geographic and ethnic populations [6], likely due to genetic and environmental influences on dental development [7,8]. While much of the current knowledge derives from studies focusing on Asian [9,10] and European [11,12] subpopulations, comparative investigations involving multiple populations remain limited [6]. A significant research gap exists regarding Central American populations, where studies are scarce despite their potential to reveal region-specific anatomical patterns. Addressing this gap could enhance clinicians’ understanding of population-based anatomical variations, ultimately improving region-specific endodontic treatment strategies.

Jamaica and Portugal present compelling case studies for exploring such variations due to their distinct genetic, cultural, and environmental contexts. Jamaica’s population, shaped by African, indigenous, and European ancestries through migration and colonization [13], contrasts with Portugal’s predominantly European ancestry [14]. Additionally, and according to some census reports, there are at least one or two Jamaicans living abroad for each household that is currently residing in Jamaica with the majority of the immigration to the United States of America, the United Kingdom, and Canada [15].

Investigating these populations using cone-beam computed tomography (CBCT), a tool that enables the precise assessment of root and canal morphology [16], could offer valuable insights into the anatomical variability of maxillary and mandibular premolars. By addressing the limited understanding of root canal anatomy in Central American populations, this study not only fills a critical gap in the scientific literature but also highlights the importance of tailoring endodontic diagnostic and treatment strategies to population-specific anatomical features. Such knowledge is crucial for improving clinical outcomes, reducing treatment failures, and ensuring more effective care for diverse patient populations.

The present study aimed to analyze the root and root canal configurations (primary outcomes) of maxillary and mandibular premolars from two distinct subpopulations—one from Central America (Jamaica) and the other from Europe (Portugal)—using CBCT imaging. The null hypothesis tested was that no differences would exist between the two populations regarding the number of roots, root canals, and the prevalence of C-shaped morphologies. Additionally, demographic factors such as age and sex (secondary outcomes) were considered to identify potential trends.

## 2. Materials and Methods

### 2.1. Research Protocol and Guidelines

The research protocol was reviewed and approved by the Ethics Committee of the Faculty of Dental Medicine, University of Lisbon, Portugal (registration number CE-FMDUL202422, date of approval: 1 April 2024). No CBCT scans were specifically obtained for this study, and patient identification was neither accessed nor disclosed. The CBCT data were collected by analyzing pre-existing imaging volumes, in accordance with the position statement on CBCT use [17]. This report adheres to the preferred reporting guidelines for epidemiological cross-sectional studies on root and root canal anatomy using CBCT technology [16].

### 2.2. Observers, Data Acquisition, and Samples

A single observer in Kingston, Jamaica (S.T.), and another in Lisbon, Portugal (J.N.R.M.), conducted a retrospective analysis of CBCT exams. Both observers received written guidelines detailing this study’s objectives, expected outcomes, definitions of anatomical landmarks, screening methodology, research timelines, relevant bibliographic references, and illustrative examples using sagittal CBCT views. They assessed pre-existing CBCT volumes consecutively, following a numerical or alphabetical chart order.

**Figure 1:**
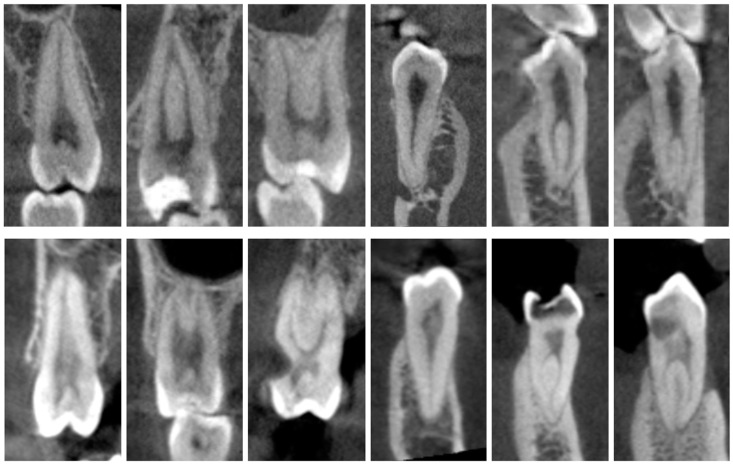
Representative CBCT images showcasing the root canal configurations in both maxillary and mandibular molars from the Jamaican (**top**) and Portuguese (**bottom**) subpopulations.

The CBCT machines, scan settings, and visualization software used in both regions were the OP 300 (Kavo, Charlotte, NC, USA) in Kingston, with an 85 µm voxel size, a voltage range of 57–90 kV, and a current range of 4–16 mA, visualized using Invivo software V5.2 (Anatomage, Santa Clara, CA, USA); and the Promax 3D (Planmeca, Helsinki, Finland) in Lisbon, with a 200 µm voxel size, 84 kV, and 15 mA, visualized using Romexis software V3.5.0 (Planmeca). All cases involved CBCT scans with a large field of view (FOV) covering the full arch. Despite differences in the CBCT scanners, settings, and visualization software, both systems offered a resolution of 200 µm or below, deemed suitable for morphological assessments [16]. Additionally, the visualization software in both regions provided comparable functionality, enabling a consistent methodology for evaluating CBCT volumes (Figure 1).

Premolars with previous root canal treatment, severe decay, immature apices, root resorption, fractures, non-restorable roots, or imaging artifacts were excluded from the analysis to ensure that the CBCT images provided clear and interpretable data for morphological assessment. These criteria were essential to minimize confounding variables and biases that could compromise the accuracy and reliability of the study findings. All included teeth were examined in three anatomical planes: coronal, sagittal, and axial. To enhance the interpretation of root canal anatomy, both observers were allowed to adjust the visualization settings within the software. The final sample comprised 2919 permanent premolars from two pre-existing imaging databases: 1380 teeth from 197 Jamaican patients (58 males and 139 females, average age 31 years) and 1539 teeth from 364 Portuguese patients (125 males and 239 females, mean age 51 years). The data were collected from health centers located in Kingston and Lisbon.

### 2.3. Outcomes

A total of 727 maxillary first premolars, 732 maxillary second premolars, 728 mandibular first premolars, and 732 mandibular second premolars were included in this study. The following data were recorded for each tooth: the number of roots (1, 2, or 3), the root canal system configuration [18], and the total number of root canals, categorized as “1 root canal”, “2 root canals”, or “3 root canals”. For mandibular premolars, the presence of C-shaped configurations was also recorded, following the criteria established by Fan et al. [2]. Additionally, demographic information, including sex and age, was recorded for each tooth.

### 2.4. Intra- and Inter-Rater Reliability Measurements

Both intra- and inter-rater reliability tests were performed to assess the consistency of the observations. For the inter-rater reliability test, CBCT sagittal view images of 40 maxillary and mandibular premolars were evaluated by both observers (S.T. and J.N.R.M.). The results were compared using Cohen’s kappa test and the percentage of agreement. For the intra-rater reliability test, each observer independently assessed 50 premolars from their respective region twice, with a one-month interval between evaluations. The results from the two assessments were compared using Cohen’s kappa test. A minimum acceptable threshold of 0.61 for both the ICC and Cohen’s kappa values was set, indicating substantial agreement [19]. If this threshold was not met, the observers were asked to review the study protocol and reassess the datasets.

### 2.5. Statistical Analysis

The primary outcomes of this study were root canal configuration and the number of roots, while the predictive variables included geographic region, sex, and age subgroups. The proportion of each morphology within each premolar group was calculated, along with the corresponding 95% confidence intervals (lower and upper limits) (IBM SPSS Statistics v.22; Chicago, IL, USA). The Z-test for proportions test was used to assess differences between the groups of interest for each premolar type. A *p*-value of <0.05 was considered statistically significant.

## 3. Results

The inter-rater reliability test showed a kappa coefficient of 0.705 (asymptotic standard error = 0.081) and a percentage of agreement of 77.5%. For intra-rater reliability, the kappa values were 0.859 (asymptotic standard error = 0.068) for S.T. and 0.920 (asymptotic standard error = 0.061) for J.N.R.M., indicating strong consistency in the observations.

The Jamaican subpopulation had a significantly higher number of roots across all tooth groups compared to the Portuguese subpopulation (*p* < 0.05) (Table 1). Specifically, the prevalence of a third root in maxillary premolars was higher among Jamaicans, with 5.5% [95% CI: 3.3–8.4%] in first premolars and 3.2% [95% CI: 1.6–5.7%] in second premolars, compared to 2.9% [95% CI: 1.5–5.1%] and 0% [95% CI: 0–0.9%] in the Portuguese population, respectively. Vertucci Type I was more common in the European group, while Vertucci Type V was more prevalent in the Central American group. Interestingly, Type VIII was observed in 6.1% [95% CI: 3.8–9.1%] of maxillary first premolars in the Jamaicans, but only in 0.8% [95% CI: 0.2–2.3%] of the Portuguese. Overall, the Central American group showed a higher frequency of three-canal configurations compared to the Portuguese group (Table 1). Mandibular first premolars in both populations showed similar proportions of C-shaped configuration (1.4% [95% CI: 0.5–3.3%] in Jamaicans and 1.8% [95% CI: 0.7–3.7%] in Portuguese individuals) (*p* > 0.05). For mandibular second premolars, the proportions were 1.7% [95% CI: 0.6–3.8%] in Jamaicans and 0.8% [95% CI: 0.1–2.2%] in Portuguese patients, also without significant differences (*p* > 0.05).

Sex differences were observed across all premolar groups, with males having a higher number of roots and root canals than females. The most significant differences were seen in maxillary first premolars, where 8.4% [95% CI: 5.2–12.6%] of males had three roots and 12.1% [95% CI: 8.3–16.9%] had three canal configurations, compared to 2.0% [95% CI: 0.9–3.7%] and 3.3% [95% CI: 1.9–5.3%] in females (*p* < 0.05) (Table 2). Age also influenced the outcomes, with individuals younger than 35 years showing a significantly higher proportion of three-rooted canal configurations across all premolar groups compared to those aged 35 years or older (*p* < 0.05) (Table 3).

## 4. Discussion

Human anatomy varies significantly across populations due to a combination of genetic, environmental, and evolutionary factors, resulting in distinct physiological and structural traits worldwide. For example, individuals of African descent generally have higher bone mineral density compared to European and Asian populations, which is linked to a lower risk of fractures in these groups [20]. Craniofacial morphology also differs between populations: individuals from colder climates, such as Northern Europe, often have narrower nasal structures, while those from tropical regions typically have broader noses, adapted to meet specific airflow needs [21]. Additionally, skin thickness and pigmentation are influenced by ultraviolet radiation exposure, with higher melanin levels in equatorial regions offering increased protection against UV damage [22]. Populations living at high altitudes, such as the Andeans, exhibit enhanced lung capacity to better utilize oxygen in low-oxygen environments [23]. These anatomical differences underscore humanity’s remarkable ability to adapt to diverse environments, emphasizing the need for regional considerations in medical research. Similar regional variations are evident in dental anatomy, particularly in the prevalence of C-shaped canal configurations in mandibular second molars, which are more common in Asian populations compared to non-Asian groups [24,25]. In the present study, significant regional differences were observed in the number of roots and root canals in premolars. However, no difference was observed in the prevalence of C-shaped configurations in mandibular premolars, partially rejecting the null hypothesis.

The present study revealed a higher proportion of single roots and single root canals (Vertucci Type I) across all premolar groups in the Portuguese subpopulation. In contrast, the Jamaican subpopulation exhibited a clear tendency towards two- and three-root morphologies, as well as Vertucci Type VIII configuration or other patterns involving three root canals (Table 1). These findings are consistent with previous research. For example, the prevalence of a three-rooted configuration in maxillary first premolars was reported as 7.3% in Jamaica and 2.0% in Mexico [6], compared to 2.6% in Spain [11], 2.0% in Portugal [6], 1.2% in Germany [12], 1.0% in Turkey [26], 0.5% in China [10], and 0% in Korea [27]. A similar pattern was observed in maxillary second premolars, where the prevalence of three roots was 3.7% in Jamaica and 2.0% in Mexico [6], compared to 1.6% in Spain [11], 0.4% in Germany [12], and 0% in Turkey [26], China [10], and Korea [27]. A comparable trend was observed in maxillary second premolars and both mandibular premolars, with Central American subpopulations exhibiting higher proportions of multi-rooted teeth compared to other global regions [6,12,26,27].

The Jamaican subpopulation predominantly exhibited Vertucci Type V root canal configurations across all premolar groups, with Type V being the most common in both maxillary and mandibular teeth. In contrast, the Portuguese subpopulation showed a higher prevalence of Types II and IV in maxillary premolars, while Type V was more frequently observed in mandibular premolars. These findings are in line with previous studies from other European populations, including those in Spain [11] and Germany [12]. However, distinct differences were noted in Turkey [26], where Type II was the most common configuration in maxillary premolars, while Type V dominated mandibular premolars. A similar trend was seen in China [10], where Types III and IV were more prevalent in maxillary premolars. These variations in root canal configurations could be indicative of specific anatomical traits within populations or may reflect the adaptive nature of the root canal system, which is influenced by both physiological and pathological factors [28,29]. Furthermore, when examining the total number of root canals in the premolars, the Jamaican subpopulation was found to exhibit a higher frequency of multi-canal systems. This observation aligns with findings from other global studies, which report fewer multi-canal configurations in populations from different regions [6,10,11,26].

Human populations display distinct variations in tooth morphology, shaped by a combination of genetic, environmental, and evolutionary factors. Research in dental anthropology and forensic odontology has highlighted specific patterns in tooth characteristics across different populations. For instance, studies suggest that individuals of African descent generally have larger teeth compared to those of European or Asian descent, a difference often attributed to ancestral dietary habits and environmental conditions. Molars in African populations typically exhibit more prominent cusps and well-defined grooves, with the “Y” pattern on mandibular molars being a common feature. This pattern is marked by a distinct arrangement of cusps [30]. Furthermore, anthropological studies have documented a higher prevalence of multiple roots in various teeth, a characteristic observed across several populations [31]. These traits may help explain the findings in the Central American subpopulation, where the Jamaican population, influenced by significant African ancestry, demonstrates similar morphological features.

For the secondary outcomes, males generally exhibited a greater number of roots and root canals compared to females, a finding that is consistent with previous studies [6]. This difference is commonly attributed to sexual dimorphism, with males exhibiting larger teeth that provide greater space for complex root and canal configurations. This trait is believed to be influenced by genetic factors, such as the presence of the Y chromosome, which promotes enhanced enamel and dentin development during odontogenesis [32,33]. Additionally, younger patients demonstrated a higher prevalence of three-root-canal configurations compared to older individuals, which contrasts with findings from some earlier studies [6]. These differences can be explained by the age-related deposition of secondary dentin, which progressively narrows the root canal lumen and may obscure accessory canals or complex configurations over time. Such calcifications, particularly in premolars with intricate anatomies, are more prominent in older individuals, making the identification of these features more challenging in CBCT imaging [28,29]. Variations in study designs, sample demographics, and imaging protocols also complicate direct comparisons between studies. These findings suggest that demographic factors, including sex and age, must be considered in clinical practice and research to improve the diagnosis and management of complex root canal systems.

Understanding variations in premolar morphology is essential for optimizing clinical outcomes, as these differences can significantly impact treatment planning and prognosis. The findings of this study underscore the critical need for clinicians to anticipate anatomical complexities, particularly in populations with a higher prevalence of multi-rooted and multi-canal systems, such as Jamaicans. These anatomical variations can influence endodontic procedures, requiring additional time, skill, and resources to locate and treat all canals effectively. Neglecting to address these complexities may result in untreated canals, persistent infections, periapical pathology, and, ultimately, compromised treatment success [34]. As demonstrated in this study, CBCT imaging is a vital tool for the preoperative identification of anatomical complexities, particularly in maxillary first premolars, which often present with three-root configurations. The integration of CBCT technology into clinical workflows enables more precise diagnostics, improves the predictability of outcomes, and reduces the likelihood of procedural errors. Additionally, CBCT may be used not only for morphological assessment but also to understand how canal architecture influences treatment efficacy [35]. In a wider context, these findings highlight the importance of incorporating population-specific anatomical data into clinical practice and dental education. Dentists, particularly those in urban areas with diverse, immigrant populations, should be well-equipped with knowledge of the anatomical characteristics associated with various ethnic groups. Furthermore, the routine use of CBCT technology should be promoted, particularly for cases where anatomical challenges are suspected. While cost considerations may present obstacles, the benefits of improved diagnostic accuracy, enhanced treatment outcomes, and long-term patient satisfaction make the integration of CBCT into everyday practice a valuable investment.

The strengths of the present study include the use of CBCT for precise, three-dimensional assessment of root and canal morphology, a large sample size of over 2919 premolars from two distinct subpopulations that enhances the reliability and generalizability of the findings, and the rigorous observer training and reliability assessments to ensure accuracy and consistency in data collection. However, certain limitations should be considered. The retrospective design of this study limits control over potential confounding factors, such as socioeconomic influences, demographic characteristics (such as age), or external dental habits, which may indirectly impact dental health or study outcomes. Another limitation of this study is its retrospective design, which relied on pre-existing CBCT datasets. Additionally, the lack of broader population comparisons, such as African or Asian subpopulations, limits the generalizability of the findings to a global context. Although variability in voxel size and visualization software between the CBCT equipment used in the two regions could introduce potential bias, efforts were made to minimize this through protocol standardization and cross-referencing of imaging parameters, ensuring that the resolution of all scans remained within the acceptable range for reliable morphological assessments [16]. Moreover, the exclusion of teeth with prior treatments or pathology may reduce the generalizability of the findings to the broader population. Finally, this study focused exclusively on two subpopulations, underscoring the need for future research to explore anatomical variations in other regions, such as Africa and Oceania, to further expand our understanding of global dental morphology.

## 5. Conclusions

This study reveals significant geographic and ethnic differences in the root and root canal morphology of premolars between Jamaican (Central America) and Portuguese (Europe) subpopulations, shedding light on the diversity of dental anatomy across populations. Jamaican individuals were found to have a higher prevalence of multi-rooted teeth and more complex canal systems in all premolar groups, particularly among males and younger patients. These findings highlight the importance of tailoring diagnostic and treatment approaches to the unique anatomical characteristics of different populations. Furthermore, this study underscores the critical role of advanced imaging technologies, such as CBCT, in endodontic practice. CBCT enables clinicians to identify complex anatomical variations more accurately, supporting better diagnosis and treatment outcomes. Incorporating such technologies into routine practice, especially in diverse and multicultural settings, can greatly enhance the precision and success of endodontic treatments.

## Figures and Tables

**Table 1:** Anatomic configuration of both maxillary and mandibular premolars in each subpopulation group.

Configuration	Maxillary Teeth				Mandibular Teeth			
	First Premolar		Second Premolar		First Premolar		Second Premolar	
	Jamaica *n* = 346	Portugal *n* = 381	Jamaica *n* = 343	Portugal *n* = 389	Jamaica *n* = 346	Portugal *n* = 382	Jamaica *n* = 345	Portugal *n* = 387
Number of roots ^1^								
1 root	89 (25.7%) *	178 (46.7%) *	214 (62.4%) *	368 (94.6%) *	321 (92.8%)	380 (99.5%)	337 (97.7%)	387 (100%)
2 roots	238 (68.8%) *	192 (50.4%) *	118 (34.4%) *	21 (5.4%) *	25 (7.2%) *	2 (0.5%) *	6 (1.7%) *	- (0%) *
3 roots	19 (5.5%)	11 (2.9%)	11 (3.2%) *	- (0%) *	- (0%)	- (0%)	2 (0.6%)	- (0%)
Root canal configuration								
Type I (1-1)	9 (2.6%)	15 (3.9%)	98 (28.6%)	150 (38.6%)	222 (64.2%)	307 (80.4%)	308 (89.2%)	371 (95.9%)
Type II (2-1)	-	62 (16.3%)	-	112 (28.8%)	-	4 (1.0%)	-	5 (1.3%)
Type III (1-2-1)	27 (7.8%)	-	54 (15.7%)	9 (2.3%)	25 (7.2%)	20 (5.2%)	3 (0.9%)	4 (1.0%)
Type IV (2-2)	2 (0.6%)	264 (69.3%)	-	72 (18.5%)	-	8 (2.1%)	-	1 (0.2%)
Type V (1-2)	282 (81.5%)	1 (0.3%)	174 (50.7%)	16 (4.1%)	90 (26.0%)	39 (10.3%)	23 (6.7%)	6 (1.6%)
Type VI (2-1-2)	-	18 (4.7%)	-	27 (6.9%)	-	-	-	-
Type VII (1-2-1-2)	2 (0.6%)	-	3 (0.9%)	-	-	2 (0.5%)	-	-
Type VIII (3-3)	21 (6.1%)	3 (0.8%)	11 (3.2%)	-	9 (2.6%)	-	11 (3.2%)	-
Other 2 root canals	-	-	-	3 (0.8%)	-	-	-	-
Other 3 root canals	3 (0.8%)	18 (4.7%)	3 (0.9%)	-	-	2 (0.5%)	-	-
Number of root canals ^1^								
1 root canal	9 (2.6%)	15 (3.9%)	98 (28.6%) *	150 (38.6%) *	222 (64.2%) *	307 (80.4%) *	308 (89.2%)	371 (95.9%)
2 root canals ^2^	313 (90.5%)	345 (90.6%)	231 (67.3%)	239 (61.4%)	115 (33.2%) *	73 (19.1%) *	26 (7.6%)	16 (4.1%)
3 root canals ^3^	24 (6.9%)	21 (5.5%)	14 (4.1%) *	- (0%) *	9 (2.6%) *	2 (0.5%) *	11 (3.2%) *	- (0%) *

^1^ Only the “Number of roots” and “Number of root canals” were subjected to statistical comparisons; ^2^ All Vertucci configuration types with maximum 2 canals combined; ^3^ All Vertucci configuration types with maximum 3 canals combined; * Differences between region groups (*p* < 0.05) within the same tooth group.

**Table 2:** Number of root and root canals of both maxillary and mandibular premolars according to sex group.

Configuration	Maxillary Teeth				Mandibular Teeth			
	First Premolar		Second Premolar		First Premolar		Second Premolar	
	Male *n* = 239	Female *n* = 488	Male *n* = 249	Female *n* = 483	Male *n* = 243	Female *n* = 485	Male *n* = 234	Female *n* = 498
1 root	61 (25.5%) *	206 (42.2%) *	191 (76.7%)	391 (81.0%)	226 (93.0%)	475 (97.9%)	230 (98.2%)	494 (99.2%)
2 roots	158 (66.1%)	272 (55.8%)	52 (20.9%)	87 (18.0%)	17 (7.0%) *	10 (2.1%) *	2 (0.9%)	4 (0.8%)
3 roots	20 (8.4%) *	10 (2.0%) *	6 (2.4%)	5 (1.0%)	- (0%)	- (0%)	2 (0.9%)	- (0%)
1 root canal	2 (0.9%) *	22 (4.5%) *	60 (24.1%) *	188 (38.9%) *	163 (67.1%)	366 (75.5%)	216 (92.3%)	463 (93.0%)
2 root canals ^1^	208 (87.0%)	450 (92.2%)	182 (73.1%)	288 (59.6%)	75 (30.8%)	113 (23.3%)	13 (5.6%)	29 (5.8%)
3 root canals ^2^	29 (12.1%) *	16 (3.3%) *	7 (2.8%)	7 (1.5%)	5 (2.1%)	6 (1.2%)	5 (2.1%)	6 (1.2%)

^1^ All Vertucci configuration types with maximum 2 canals combined; ^2^ All Vertucci configuration types with maximum 3 canals combined; * Differences between sex groups (*p* < 0.05) within the same tooth group.

**Table 3:** Number of root canals of both maxillary and mandibular premolars according to age group.

Configuration	Maxillary Teeth				Mandibular Teeth			
	First Premolar		Second Premolar		First Premolar		Second Premolar	
	<35 years *n* = 308	≥35 years *n* = 419	<35 years *n* = 323	≥35 years *n* = 409	<35 years *n* = 276	≥35 years *n* = 452	<35 years *n* = 284	≥35 years *n* = 448
1 root canal	13 (4.2%)	11 (2.6%)	106 (32.8%)	142 (34.7%)	183 (66.3%)	346 (76.5%)	254 (89.4%)	425 (94.9%)
2 root canals ^1^	266 (86.4%)	392 (93.6%)	203 (62.9%)	267 (65.3%)	85 (30.8%)	103 (22.8%)	19 (6.7%)	23 (5.1%)
3 root canals ^2^	29 (9.4%) *	16 (3.8%) *	14 (4.3%) *	- (0%) *	8 (2.9%) *	3 (0.7%) *	11 (3.9%) *	- (0%) *

^1^ All Vertucci configuration types with maximum 2 canals combined; ^2^ All Vertucci configuration types with maximum 3 canals combined; * Differences between age groups (*p* < 0.05) within the same tooth group.

## Data Availability

The original contributions presented in this study are included in the article. Further inquiries can be directed to the corresponding author.
